# Reply: The Pragmatic Approach or Simply the Better Approach?

**DOI:** 10.1016/j.jacadv.2025.102134

**Published:** 2025-09-10

**Authors:** Subaina Khalid, Julia Ma, Sarosh Janjua, Cynthia C. Taub, Courtney L. Maxey-Jones

**Affiliations:** a Department of Medicine, SUNY Upstate, Syracuse, New York, USA; b CNY Medical Services, PLLC, Baldwinsville, New York, USA

Given the complexity of critically ill cardiac patients, we discussed and agreed on the importance of a collaborative approach to ensure comprehensive, high-quality care.^1^ Recent data from an American Heart Association-led national survey revealed substantial variation in how cardiac intensive care units (CICUs) are staffed, with fewer than 15% having dedicated cardiac intensivists.^2^

In hospitals without consistent cardiac intensivist staffing, collaborative care models—where general intensivists support or comanage CICU patients—have been recommended as a practical approach that modulates responsibility based on patient acuity.^3^ Shared care is extendable to multiple health care settings, including community hospitals, hybrid ICUs, or those with limited availability of intensivists that may be supplemented with telemedicine. However, these models are not equivalent to dedicated subspecialist care. Increased acuity and complexity of CICU patients often require expertise that extends beyond general cardiology or critical care training. Observational studies have shown that closed CICUs staffed by cardiac intensivists are associated with improved outcomes, including reduced mortality and shorter ICU stay.^4^

While we agree that general intensivists primarily staff CICUs nationwide, we advocate for specialized cardiac critical care (CCC) accreditation at Level 3 centers due to the increasing complexity of CICU patients. We commend your program for extensively training fellows in pulmonary artery catheter placement/interpretation and cardiac postoperative care/extracorporeal membrane oxygenation. However, there is variable case complexity and exposure to critically ill cardiology patients in critical care medicine training programs across the country, and it remains rare for critical care medicine trainees to gain both exposure to and comfort with pulmonary artery catheter placement, mechanical circulatory support devices, and cardiac postoperative care.

Some have proposed integrating focused critical care training into general cardiology and critical care fellowships to reduce the need for dedicated cardiac intensivists. This would allow fellows to obtain non-Accreditation Council for Graduate Medical Education (ACGME) CCC certifications, enabling practice across different acuity levels (Level 1/2/3 centers).^5^ However, without ACGME accreditation, these qualifications vary and lack widespread acknowledgment, limiting their value and appeal.

It may be worth advocating for American Board of Internal Medicine (ABIM) recognition of CCC as a subspecialty. Additionally, non-ACGME-accredited CCC training may be sufficient in Level 1 and 2 centers, while formal ACGME-accredited training in CCC may be required at Level 3 centers. Achieving this would require extensive collaboration, particularly between ACGME and ABIM, but similar efforts have succeeded before.
